# Repetitive part of the banana (*Musa acuminata*) genome investigated by low-depth 454 sequencing

**DOI:** 10.1186/1471-2229-10-204

**Published:** 2010-09-16

**Authors:** Eva Hřibová, Pavel Neumann, Takashi Matsumoto, Nicolas Roux, Jiří Macas, Jaroslav Doležel

**Affiliations:** 1 Laboratory of Molecular Cytogenetics and Cytometry, Institute of Experimental Botany, Sokolovská 6, Olomouc, CZ-77200, Czech Republic; 2 Biology Centre ASCR, Institute of Plant Molecular Biology, Branišovská 31, České Budĕjovice, CZ-37005, Czech Republic; 3 National Institute of Agrobiological Sciences, Kannondai, Tsukuba, Ibaraki 305-8602, Japan; 4 Commodities for Livelihoods Programme, Bioversity International, Parc Scientifique Agropolis II, 34397 Montpellier Cedex 5, France

## Abstract

**Background:**

Bananas and plantains (*Musa *spp.) are grown in more than a hundred tropical and subtropical countries and provide staple food for hundreds of millions of people. They are seed-sterile crops propagated clonally and this makes them vulnerable to a rapid spread of devastating diseases and at the same time hampers breeding improved cultivars. Although the socio-economic importance of bananas and plantains cannot be overestimated, they remain outside the focus of major research programs. This slows down the study of nuclear genome and the development of molecular tools to facilitate banana improvement.

**Results:**

In this work, we report on the first thorough characterization of the repeat component of the banana (*M. acuminata *cv. 'Calcutta 4') genome. Analysis of almost 100 Mb of sequence data (0.15× genome coverage) permitted partial sequence reconstruction and characterization of repetitive DNA, making up about 30% of the genome. The results showed that the banana repeats are predominantly made of various types of Ty1/*copia *and Ty3/*gypsy *retroelements representing 16 and 7% of the genome respectively. On the other hand, DNA transposons were found to be rare. In addition to new families of transposable elements, two new satellite repeats were discovered and found useful as cytogenetic markers. To help in banana sequence annotation, a specific *Musa *repeat database was created, and its utility was demonstrated by analyzing the repeat composition of 62 genomic BAC clones.

**Conclusion:**

A low-depth 454 sequencing of banana nuclear genome provided the largest amount of DNA sequence data available until now for *Musa *and permitted reconstruction of most of the major types of DNA repeats. The information obtained in this study improves the knowledge of the long-range organization of banana chromosomes, and provides sequence resources needed for repeat masking and annotation during the *Musa *genome sequencing project. It also provides sequence data for isolation of DNA markers to be used in genetic diversity studies and in marker-assisted selection.

## Background

Bananas and plantains (*Musa *spp.) are perennial giant herbs grown in humid tropical and subtropical regions. Their annual production exceeds 100 million tons, out of which almost 90% is targeted for local and national markets [1]. Cultivated bananas are parthenocarpic, seed-sterile, vegetatively-propagated diploid, triploid and tetraploid clones. Most of them are hybrids between two diploid (2n = 2x = 22) species *M. acuminata *and *M. balbisiana *[2] with the A and B genomes respectively. The production of bananas is threatened by many diseases and pests, but the clonal nature, seed sterility and the lack of knowledge on the origin of cultivated clones hampers breeding of improved cultivars. It is expected that the use of molecular tools will speed up banana germplasm improvement. Sadly, although the socio-economic importance of bananas and plantains cannot be questioned, *Musa *remains outside the focus of major research programs and must be considered an under-researched crop.

This situation is reflected by a limited knowledge of the banana nuclear genome, even though it is relatively small (1C ~ 600 Mbp) [3,4]. It has been estimated that about 55% of the genome is made of various DNA repeats [5], but only a limited number of repetitive DNA sequences has been characterized. Valárik *et al*. (2002) described twelve *Radka *repeats [6], representing partial sequences of various mobile elements and rRNA genes. Other characterized sequences included a *Copia*-like element [7,8], a species-specific element Brep-1 [9,10] and a Ty3/*gypsy*-like retrotransposon *monkey *[11]. In order to identify more repeats, Hřibová *et al. *[5] applied a low-Cot DNA isolation technique to characterize highly repetitive fractions of banana genome. An important step forward in dissecting the *Musa *genome was made by Cheung and Town (2007), who sequenced ends of more than 6,000 BAC (Bacterial Artificial Chromosome) clones [12]. Moreover, 62 BAC clones were completely sequenced through a Generation Challenge Programme funded project (GCP 2005-15), within the context of the Global *Musa *Genomics Consortium [13]. Nevertheless, even after these efforts, the knowledge on the repetitive part of *Musa *genome remains far from complete.

Recent introduction of the next generation sequencing methods [14] provided powerful tools to discover and characterize DNA repeats, even in complex plant genomes. For example, Macas *et al*. (2007) used the 454 technology to characterize repetitive DNA in the nuclear genome of pea (*Pisum sativum *L.) [15]. Despite the relatively small proportion of sequenced DNA relative to the whole genome (33.3 Mb or ~ 0.77% of the genome), the authors identified and characterized most types of retrotransposons and discovered thirteen new families of tandemly organized repeats. In a similar study, Swaminathan *et al*. (2007) used the 454 system to sequence 7.5% of the soybean genome [16].

This study addresses the lack of knowledge on the repetitive part of the banana genome by characterizing all major DNA repeats after massively parallel sequencing of genomic DNA of a diploid clone of *M. acuminata*. The experimental approach follows that of Macas *et al. *[15], in which all-to-all similarity comparison of 454 reads is performed to identify groups (clusters) of overlapping reads representing repetitive genomic sequences. As the number of reads in individual clusters is proportional to genomic abundance of corresponding repeats, this information can be used for quantitative analysis of repetitive genome landscape. In addition, consensus sequences of the repeated elements can be obtained by assembling the reads within clusters. We also demonstrate how databases of 454 reads sorted according to the type of repeats can be used to identify and classify repeats in BAC sequences. Finally, the large set of sequences obtained in this study provides a unique source of molecular markers potentially useful in genome mapping, anchoring physical maps, analyzing genetic diversity and for phylogenetic studies.

## Results and Discussion

A diploid clone *M. acuminata *cv. 'Calcutta 4' was chosen for sequencing as it has been used extensively as a model genotype in previous molecular studies [5,6,12,17-19]. Moreover, this clone is being used in various banana breeding programs as a source of diseases resistance [20,21]. A sequencing run of nuclear DNA on the GS FLX platform (454 Life Sciences/Roche) resulted in 477,699 reads with average length of 206 bp, providing a total of 98,538,911 bp of sequence data. Considering genome size of 'Calcutta 4' (1C = 623 Mbp) [4], this represents 15.7% of the genome. The sequencing reads were clustered based on their similarity and all clusters containing at least 20 reads (roughly representing 0.01% of the genome) were further investigated.

### LTR-retrotransposons

**Figure 1:**
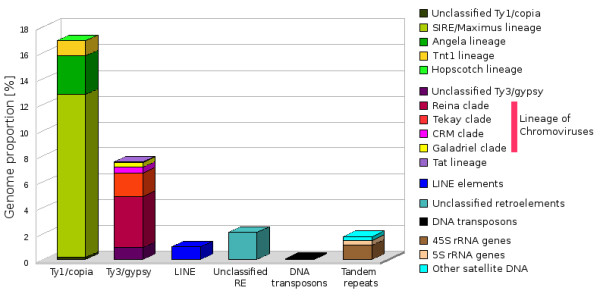
**Genome proportion of major groups of repetitive sequences identified in banana 454 data**.

**Figure 2:**
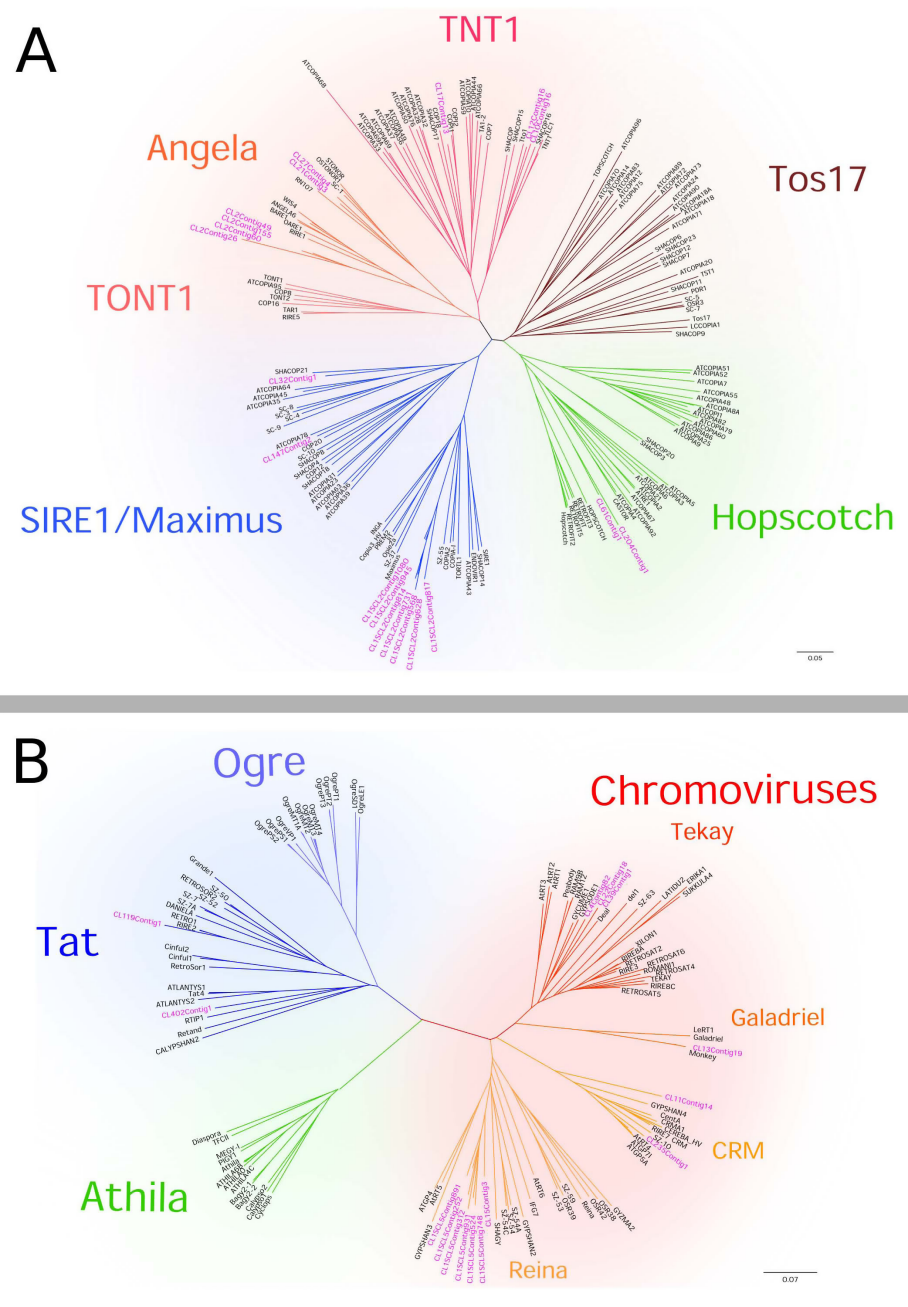
**Phylogenetic analysis of *Musa *retrotransposons based on RT sequences**. Unrooted phylogenetic trees of Ty1/*copia *elements (A) and Ty3/*gypsy *elements (B). Names of the contigs assembled from 454 reads are printed in purple. Classification of the Ty3/*gypsy *lineages and chromoviral clades was done according to [30-32]. Major lineages of Ty1/*copia *elements were named according to a selected representative of each group.

The most abundant DNA sequences found in the banana genome were LTR-retrotransposons. Out of them, Ty1/*copia *represented more than 16% of the genome while the Ty3/*gypsy *elements represented about 7% of the genome (Figure 1). This is an interesting observation as the available data from other sequencing projects indicate prevalence of Ty3/*gypsy *retrotransposons in plant nuclear genomes [15,22-24]. In order to get insight into the diversity of banana LTR-retrotransposons, we performed phylogenetic analysis based on a comparison of their reverse transcriptase domains. This work revealed that while the more abundant Ty1/*copia*-like elements were represented by four distinct evolutionary lineages (Figure 2A), vast majority of Ty3/*gypsy*-like elements belonged to a single evolutionary lineage of chromoviruses [25] (Figure 2B).

**Figure 3:**
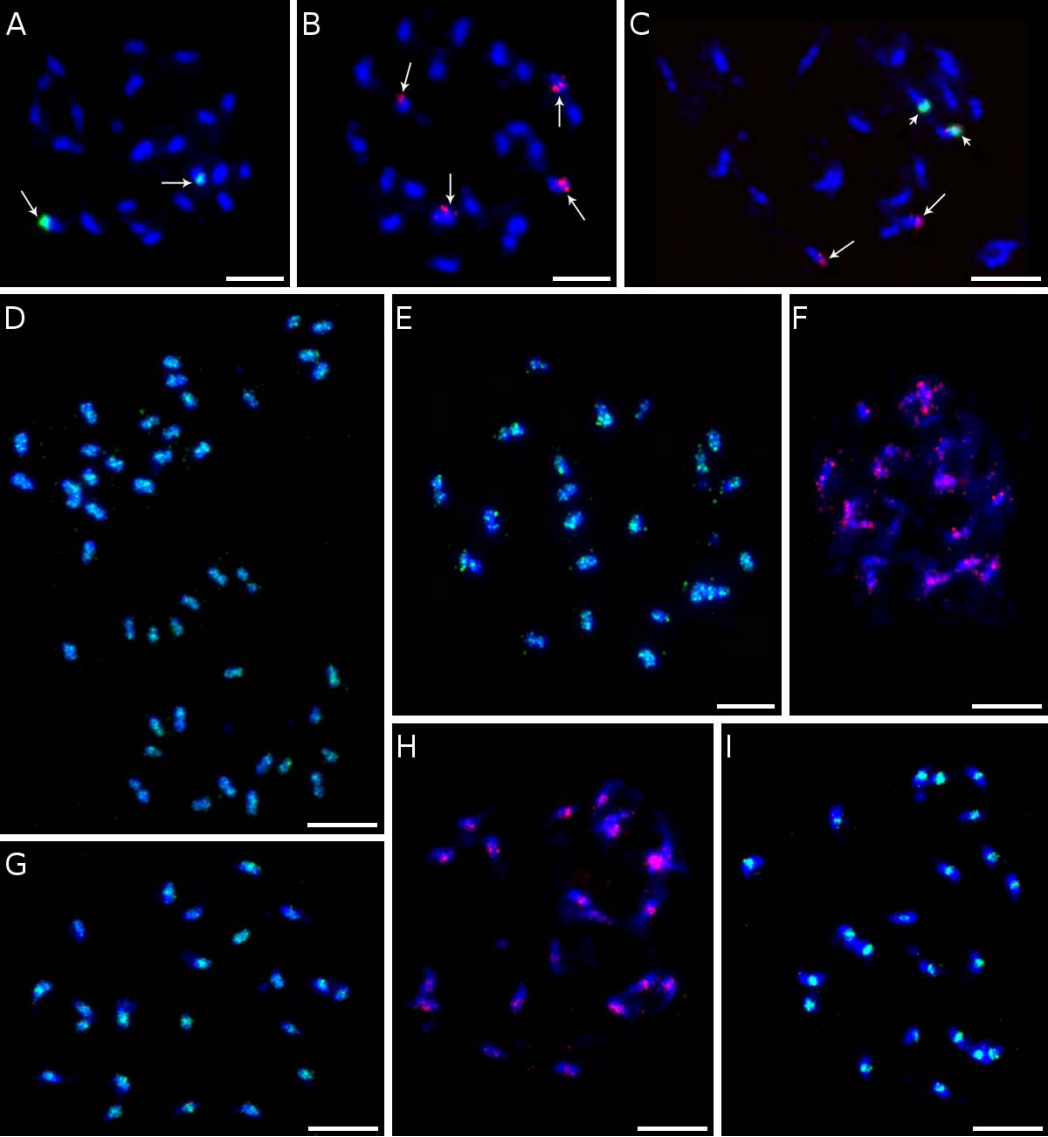
**Genomic distribution of different types of DNA repeats**. Mitotic metaphase spreads of *M. acuminata *cv. 'Calcutta 4' (2n = 22) after FISH with probes for various repeats. The chromosomes were counterstained with DAPI (blue). Bar = 5 μm. (A) Tandem repeat CL18 (green signal) formed a cluster on one pair of chromosomes (long arrows). (B) Tandem repeat CL33 (red signal) localized on two pairs of chromosomes (long arrows). (C) Simultaneous hybridization of probes for CL18 (green signal) and CL33 (red signal, long arrows) revealed co-localization of both satellites on one pair of chromosomes (short arrows). (D) Two metaphase plates after FISH with a probe for CL1SCL2Contig1080 - banana retrotransposon belonging to SIRE/Maximus lineage (green). Uneven genomic distribution with clusters dispersed on all chromosomes is obvious. (E) Similar genomic distribution was found for banana retroelement related to the Angela lineage (CL2Contig49). (F) Banana retrotransposon belonging to Tnt1 lineage (CL10Contig16, red color) gave weak signals preferentially localized in distal parts of chromosomes (long arrows). (G) The most abundant type of Ty3/*gypsy*-like element of the Reina lineage (CL1SCL5Contig891) localized preferentially to centromeric or peri-centromeric regions of all chromosomes (green signals). (H) Also the Ty3/*gypsy*-like element related to Tekay evolutionary lineage (CL4Contig82) clustered in centromeric or peri-centromeric regions of all chromosomes. (I) A probe derived from LINE element (CL1SCL8Contig452) localized in the centromeric regions of all chromosomes (green signals).

About 74% of identified Ty1/*copia *sequences belonged to the SIRE/Maximus lineage [26,27], representing almost 13% of the genome. The remaining Ty1/*copia *elements belonging to Angela, Tnt1 and Hopscotch lineages [27-29] represented only about 3.0, 1.0 and 0.2% of the genome, respectively. Interestingly, fluorescence *in situ *hybridization (FISH) on mitotic chromosomes revealed that elements from distinct evolutionary lineages have different patterns of genomic distribution. The elements from the SIRE/Maximus and Angela lineages were concentrated in several discrete clusters on all chromosomes (Figures 3D, E) and the elements from the Tnt-1 lineage gave only weak signals preferentially localized in distal parts of mitotic chromosomes (Figure 3F). Elements belonging to the Hopscotch lineage were not tested for the distribution because of their very low proportion in the genome.

Ty3/*gypsy*-like retrotransposons showed relatively low degree of phylogenetic diversity and most of them belonged to the lineage of chromoviruses. This single lineage comprised about 87% of Ty3/*gypsy *elements identified in this study, thus greatly outnumbering elements from the Tat lineage, which included all other Ty3/*gypsy *elements identified in the banana genome. The chromoviral sequences could be classified into four clades: Galadriel, Tekay, Reina and CRM [30-32]. The most abundant chromoviral clade was Reina, which involved more than half of all chromoviral sequences, making up about 4% of the banana genome. Many elements belonging to this clade appeared to be non-autonomous as they lacked parts of RT-coding domain (data not shown). Members of the Tekay clade were found to be the second most abundant group of chromoviruses, reaching about 2% of the genome. Sequences from the Galadriel clade corresponded to the retrotransposon *monkey*, which has been identified earlier in the banana genome [11]. The consensus sequences of the *monkey *retrotransposon assembled from our 454 data as a 5880 bp fragment showed 95% similarity to the *monkey *element described by Balint-Kurti *et al*. (2000) [11]. Previous estimates of the copy number using slot-blot analysis indicated that *monkey *constituted about 0.2 - 0.5% of the *M. acuminata *genome [11] and are on line with our estimates based on the proportion of monkey-derived sequences in 454 reads (Additional file 1). Although the *monkey *was supposed to be the most abundant repetitive element in banana [5], our data showed that several other families of retroelements account for much larger parts of the genome. The CRM clade sequences occupied a similar fraction of the genome as those from the Galadriel clade. Although being members of the same evolutionary lineage, banana chromoviruses from distinct clades partly differed in their chromosomal distribution. Contrary to *monkey*, which preferentially localized in secondary constrictions [11], members of other clades occupied mostly pericentromeric regions and some additional loci in distal parts of all chromosomes (Figures 3G, H).

### Non-LTR retrotransposons and DNA transposons

Compared to LTR-retrotransposons, non-LTR retrotransposons and DNA transposons were found relatively rare (Additional file 1). Within the clusters that represented at least 0.01% of the genome, only one cluster of LINE sequences [33] and two clusters of DNA transposons were identified. The LINE elements were estimated to constitute about 1% of the banana genome. FISH with a probe derived from reverse transcriptase domain of a LINE-like element, resulted in dot signals in centromeric regions on all chromosomes (Figure 3I). DNA transposons identified in this work included elements that showed similarity to transposons belonging to the hAT superfamily [34]. FISH with a probe derived from hAT-related element failed to give visible signals, most probably due to relatively small copy number. The low abundance of LINEs and DNA transposons seems to be typical for plant genomes and similar abundances were observed for example in rice, grape and maize genomes [23,24,35].

### 45S and 5S rDNA

Clusters containing 45S rDNA represented 1.12% of the genome and the 45S rDNA sequence region was reconstructed as a 7,553 bp fragment that included complete sequence of the 18S-5.8S-26S rRNA locus surrounded by parts of intergenic spacer (IGS). Moreover, based on similarity searches to BAC clone MA4_01C21 from *M. acuminata*, which was sequenced within the context of the Global *Musa *Genomics Consortium [13] and which carries 45S rDNA units, another cluster containing IGS-like sequence was identified in the 454 data.

In contrast to Balint-Kurti *et al*. (2000) whose results obtained after FISH with mitotic chromosomes indicated insertion of a part of *monkey *into 45S rDNA [11], our 454 data suggests that *monkey *is not frequently associated with the 18S-5.8S-26S rRNA gene copies. A plausible explanation for this discrepancy is that the insertion is adjacent to the 45S rDNA locus. In fact, the spatial resolution of FISH on mitotic chromosomes is not sufficient to discriminate two loci closer than 5 - 10 Mbp [36,37]. A close vicinity of the *monkey *fragment to 45S rDNA is supported by the sequence data of the BAC MA4_01C21 comprising 45S rDNA, in which a 1.5kb fragment of *monkey *was identified. However, the fragment was not inserted in the 45SrDNA or IGS sequences. The fact that this BAC comprises a chromovirus element from the Tekay lineage and the SIRE1/Maximus lineage indicates that the BAC MA4_01C21 actually encompasses a border of the 45S rDNA locus and flanking genomic sequences, characterized by sequence-heterogeneity and insertion of various mobile elements.

Similar to the 45S rRNA gene cluster, our 454 sequence data enabled reconstruction of the entire coding part of the 5S rRNA gene and its non-transcribed spacer. The 5S rDNA was found to represent 0.38% of the banana nuclear genome. Teo *et al. *[38] identified a Ty1/*copia*-like element in the 5S rDNA spacer in several banana species. The analysis of retrotransposon protein coding domains in our data confirmed that the 5S rDNA spacer contained a part of the reverse transcriptase of the Tnt1-like element.

### Tandem organized repeats

Repeat reconstruction from the 454 data led to discovery of two new tandemly organized repeats. One of them (CL33) consists of ~130 bp monomer while the CL18 repeat is characterized by ~2 kb monomer unit. FISH on mitotic chromosomes revealed clusters of signals in the subtelomeric regions of one pair of chromosomes (satellite CL18) and weak signals in telomeric region on two pairs of chromosomes (satellite CL33) (Figures 3A, B, C). Southern hybridization resulted in a ladder-like pattern typical for tandemly organized repetitive units for repeat CL33, only (not shown). The repeat CL18 gave a weak smear with a few visible bands, most likely due to partially dispersed distribution and/or poor conservation of the monomer length. A rather low copy number of CL33 and/or long repetitive unit of satellite CL18 may explain why these repeats were not identified in previous studies [5,6]. In general, the absence of more abundant tandem repeats in the banana genome may be related to its relatively small size. Satellite DNA is a typical component of subtelomeric and centromeric chromosome regions in various plant species, but they often form blocks of repeats in interstitial regions [15,39-42]. The results of this study, as well as our earlier observations [5,6] indicate that typical centromeric satellite DNA is absent in the banana genome, and that the centromeric regions are likely to be made of various types of retrotransposons.

### Identification of DNA markers

**Figure 4:**
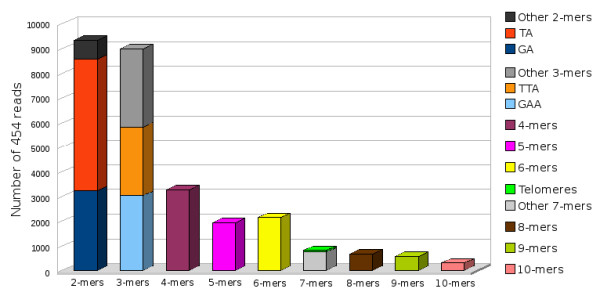
**Major groups of microsatellite DNA sequences identified in banana 454 reads**.

Following the thorough characterization of banana repetitive DNA, we screened the 454 sequences for the presence of loci potentially suitable for use as DNA markers. We focused on identification of simple sequence repeats (SSRs) and sites of insertions of transposable elements (ISBP - Insertion Site Based Polymorphism) [43]. In total, 27,946 of 454 reads containing SSRs were identified with repeat units ranging from 2 to 10 bp. The most abundant motifs were dinucleotides TA and GA and trinucleotides GAA (Figure 4). More than 11,000 reads were identified to contain potential ISBP sites, most of them carrying insertions of retrotransposons into unknown low-copy sequences. Databases containing 454 reads carrying SSR sequences and potential ISBPs were established and made publicly available on our website [44].

### Repeat identification in sequenced DNA clones

**Figure 5:**
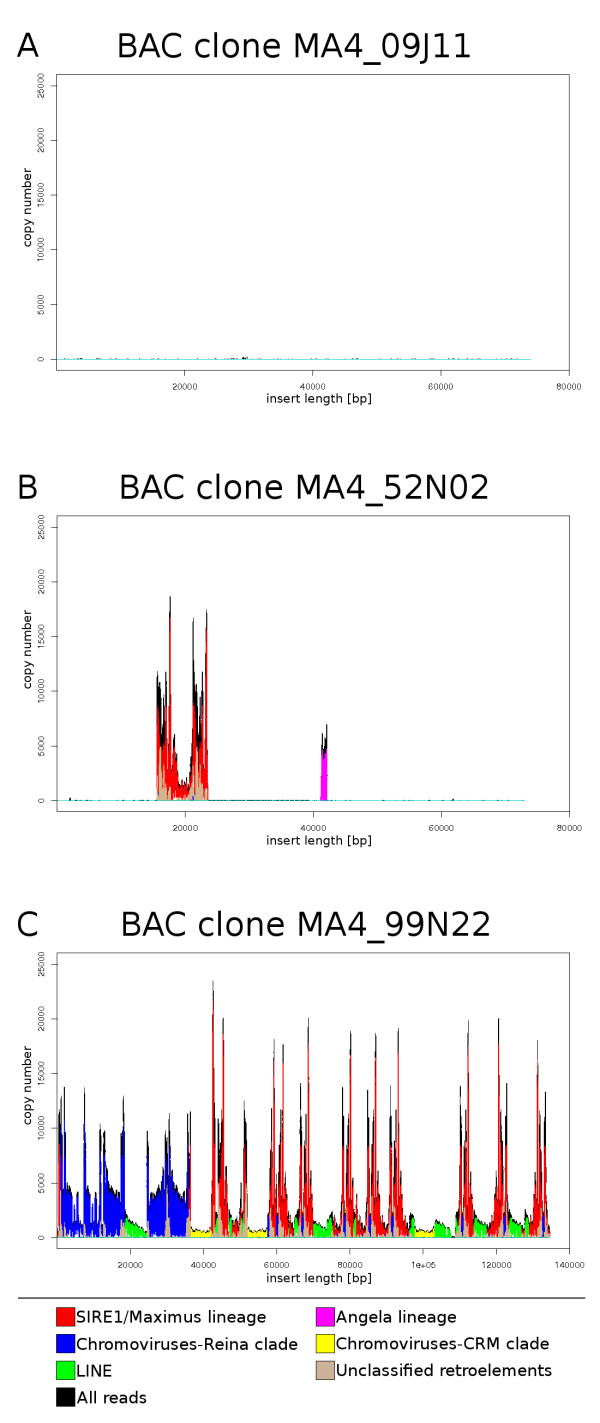
**Examples of repeat identification in *M. acuminata *BAC clones**. Nucleotide sequences of BAC inserts are represented on X axis. The plots represent genomic copy numbers of individual insert regions calculated from numbers of similarity hits to 454 read databases. The plot colors correspond to different types of the repeats. (A) A 'low-copy' BAC clone showing absence of repeats along its entire length. (B) A BAC clone with relatively long stretches of repetitive DNA. (C) A highly repetitive BAC clone with various types of repeats.

As the next step in utilizing 454 data, we took advantage of the read clustering during repeat analysis and created databases of sequence reads sorted according to their repeat of origin. These databases were then utilized for similarity-based repeat detection and classification in genomic BAC clone sequences implemented at the PROFREP server [45]. The analysis was performed for 49 BAC clones from *M. acuminata *cv. 'Calcutta 4' and for 12 BAC clones from *M. balbisiana *cv. 'Pisang Klutug Wulung' [14]. The clones were sequenced as part of the Generation Challenge Program supported project (GCP-2005-15) and were selected based on the presence of resistance gene homologs and other gene-like sequences. Indeed, out of the 49 *M. acuminata *BAC clones, only 9 clones were highly repetitive. 15 BAC clones contained a single copy sequence with a large repetitive region and the remaining BAC clones comprised single copy sequence without any detectable repetitive DNA and/or carried a very short repetitive region (Figure 5). Out of the 12 BAC clones from *M. balbisiana*, two were single copy, while the remaining 10 BAC clones carried low copy sequences mixed with large repetitive regions. The repetitive profiles of all 62 BAC clones are available as supplementary data (Additional file 2).

## Conclusions

This work represents a major advance in the analysis of the nuclear genome organization in banana, an important staple and cash crop. The application of low-depth 454 sequencing provided until now the largest amount of DNA sequence data, and enabled a detailed analysis of repetitive components of its nuclear genome. All major types of DNA repeats were characterized and *Musa *DNA repeats databases were established. The analysis of genomic distribution of selected repeats provided new data on long-range molecular organization of banana chromosomes, and a large number of loci potentially useful as DNA markers were identified. The improved knowledge and resources generated in this study will be useful in annotating the banana genome sequence, in the analysis of the evolution of the *Musa *genome, and for the study of dynamics of DNA repeats over evolutionary time scale, as well as to isolate DNA markers for use in genetic diversity studies and in marker-assisted selection.

## Methods

### 454 sequencing

*In vitro *rooted plants of *M. acuminata *cv. 'Calcutta 4' (ITC 0249) were obtained from the International Transit Centre (Bioversity International, Global *Musa *Genebank hosted by the Katholieke Universiteit, Leuven, Belgium) and grown in a greenhouse. DNA for sequencing was prepared from nuclei isolated from healthy young leaf tissues according to Zhang *et al*. (1995) [46]. Isolated nuclei were incubated with 40 mM EDTA, 0.2% SDS and 0.25 μg/μl proteinase K for 5 hours at 37°C, and DNA was purified by phenol/chloroform precipitation. The 454 sequencing was performed at the Arizona Genomics Institute (Tucson, USA) using 454 Life Sciences/Roche FLX instrument. All sequence information generated in this study are available on our website [44] and was submitted to the National Center for Biotechnology Information short read archive under accession numbers SRR057410 and SRR057411.

### Data analysis

Following a removal of linker/primer contaminations and artificially duplicated reads, the remaining 477,699 reads (average length of 206 nucleotides) were used for repeat analysis. The analysis was performed as described by Macas *et al*. (2007) [15], employing TGICL [47] and a set of custom-made BioPerl scripts for similarity-based clustering and assembly of reads. The clustering parameters used by a tclust program (part of TGICL) were set to consider pairwise similarity of two reads significant if it involved an overlap of at least 150 nucleotides with 90% or better similarity, representing at least 55% and 70% of the length of longer and shorter read respectively (OVL = 150 PID = 90 LCOV = 55 SCOV = 70). The reads within individual clusters were assembled into contigs using TGICL run with the -O '-p 80 -o 40' parameters, specifying overlap percent identity and minimal length cutoff for cap3 assembler. Repeat type identification was done using blastn and blastx [48] sequence-similarity searches of assembled contigs against GenBank, and by detection of conserved protein domains, using RPS-BLAST [49]. Tandem repeats within contig sequences were identified using dotter [50]. The classification of LTR retrotransposons into distinct lineages and clades was done using phylogenetic analyses of their RT sequences [15]. Alignment of RT sequences was carried out with ClustalX [51] and the phylogenetic trees were calculated using neighbour-joining method. The trees were drawn and edited using the FigTree program.

Microsatellite sequences were identified using Tandem Repeats Finder [52] and TRAP [53] programs, while a BioPerl script was used to identify ISBP loci [54]. Identification and classification of repetitive sequences within BAC clones was done via PROFREP web server [45] utilizing repeat-specific databases of 454 reads prepared in this study. The server performs BLAST-based searches against databases of whole-genome or repeat-specific 454 reads and generates plots of similarity hits along the query sequence (number of hits is proportional to copy number of the query in the genome).

### Preparation of probes for cytogenetic mapping

Primers specific for tandem repeats (Additional file 3) were designed from sequence contigs that carry tandem organized repetitive units. Labeled probes were prepared by PCR on *M. acuminata *'Calcutta 4' genomic DNA with biotin- and digoxigenin-labeled nucleotides. The PCR premix contained 1× PCR buffer, 1 mM MgCl_2_, 0.2 mM dNTPs, 0.2 μM primers, 0.5 U Taq polymerase (Finnzymes) and 10 - 15 ng template DNA. PCR reaction was performed as follows: initial denaturation of 3 min at 94°C followed by 30 cycles of 1 min at 94°C, 50 s at 57°C and 50 s at 72°C and final extension step 5 min at 72°C.

Specific primers were also designed for reverse transcriptase (RT) domains of different retroelements (Additional file 3). In the first step, the RT domains were amplified using PCR with a mix containing 1× PCR buffer, 1.5 mM MgCl_2_, 0.2 mM dNTPs, 0.2 μM primers, 0.5 U Taq polymerase (Finnzymes) and 10 - 15 ng template DNA. PCR products were checked by gel-electrophoresis, cleaned up using paramagnetic beads Agencourt Ampure (Beckman Coulter), cloned into TOPO vector (Invitrogen) and transformed into electro-competent *E. coli *cells. 48 recombinant clones for each retroelement were PCR amplified using M13 primers and separated on the gel electrophoresis. Clones for each RT domain were than cleaned up using ExoSAP-IT (USB Corporation), and used for Sanger sequencing to verify presence of specific RT domains in the clones. The PCR products were sequenced with BigDye Terminator 3.1 Cycle Sequencing Kit (Applied Biosystems) on ABI 3730xl DNA analyzer (Applied Biosystems). The nucleotide sequences were analyzed and edited using the Staden Package [55], and searched for similarity with the corresponding 454 contigs using BLAST [48]. Clones with the highest similarity to reconstructed contigs were PCR amplified with biotin- and digoxigenin-labeled nucleotides and used as probes for fluorescence *in situ *hybridization. Selected clones used as probes showed at least 98% similarity with the corresponding 454 sequence.

### Fluorescence in situ hybridization (FISH)

FISH was done on mitotic metaphase spreads prepared from meristem root tip cells as described by Doleželová *et al*. (1998) [56]. The hybridization mixture consisted of 40% formamide, 10% dextran sulfate in 1 × SSC and a 1 μg/ml labeled probe. The mixture was added onto slides and denatured at 80°C for 4 min. The hybridization was carried out at 37°C overnight. The sites of probe hybridization were detected using anti-digoxigenin-FITC (Roche Applied Science) and streptavidin-Cy3 (Vector Laboratories), and the chromosomes were counterstained with DAPI. The slides were examined with Olympus AX70 fluorescence microscope (Olympus) and the images of DAPI, FITC and Cy-3 fluorescence were acquired separately with a cooled high-resolution black and white CCD camera. The camera was interfaced to a PC running the MicroImage software (Olympus).

## Authors' contributions

EH isolated genomic DNA for sequencing, and performed detailed repeat analysis and their cytogenetic mapping. JM performed initial 454 read clustering/assembly and PN conducted phylogenetic classification of retrotransposon sequences. TM sequenced BAC clones. JD and JM made an intellectual contribution to the concept of the study and JD, JM and NR revised the manuscript critically for important intellectual content. All authors read and approved the final manuscript.

## Supplementary Material

Additional file 1 **Genome proportion of newly characterized banana repetitive elements**. Genome proportion of repetitive elements was estimated from the sum of genome representation values (GR) of corresponding clusters of reads. Click here for file

Additional file 2 **Repetitive profiles of sequenced BAC clones**. DNA sequence profiles of selected clones from three BAC libraries of *M. acuminata *cv. 'Calcutta 4' (MA4 and C4BAM), *M. acuminata *cv. 'Cavendish' (MAC) and MBP BAC library from *M. balbisiana *cv. 'Pisang Klutug Wulung' http://olomouc.ueb.cas.cz/dna-libraries/bananas. Click here for file

Additional file 3 **Primers used for PCR amplification of satellite DNA and different types of retrotransposons**. Click here for file

## References

[B1] Food and Agriculture Organization (FAO) http://faostat.fao.org/

[B2] Simonds NW Shepherd K The taxonomy and origins of the cultivated bananas J Linn Soc Bot 1955 55 302 312 10.1111/j.1095-8339.1955.tb00015.x

[B3] Doležel J Doleželová M Novák FJ Flow cytometric estimation of nuclear DNA amount in diploid bananas (*Musa acuminata *and *M. balbisiana*) Biol Plantarum 1994 36 351 357 10.1007/BF02920930

[B4] Bartoš J Alkhimova O Doleželová M De Langhe E Doležel J Nuclear genome size and genomic distribution of ribosomal DNA in *Musa *and *Ensete *(Musaceae): taxonomic implications Cytogenet Genome Res 2005 109 50 57 10.1159/000082381 15753558

[B5] Hřibová E Doleželová M Town CD Macas J Doležel J Isolation and characterization of the highly repeated fraction of the banana genome Cytogenet Genome Res 2007 119 268 274 10.1159/000112073 18253041

[B6] Valárik M Šimková H Hřibová E Safář J Doleželová M Doležel J Isolation, characterization and chromosome localization of repetitive DNA sequences in bananas (*Musa *spp.) Chromosome Res 2002 10 89 100 10.1023/A:1014945730035 11993938

[B7] Baurens FC Noyer JL Lanaud C Lagoda PJL A repetitive sequence family of banana (*Musa *sp.) shows homology to *Copia*-like elements J Genet Breed 1997 51 135 142

[B8] Teo CH Tan SH Othman YR Schwarzacher T The cloning of Ty 1 -copia -like retrotransposons from 10 varieties of banana (Musa Sp.) J Biochem Mol Biol Biophys 2002 6 193 201 10.1080/10258140290022329 12186754

[B9] Baurens FC Noyer JL Lanaud C Lagoda PJL Use of competitive PCR to essay copy number of repetitive elements in banana Mol Gen Genet 1996 253 57 64 10.1007/s004380050296 9003287

[B10] Baurens FC Noyer JL Lanaud C Lagoda PJL Assessment of a species-specific element (Brep 1) in banana Theor Appl Genet 1997 95 922 931 10.1007/s001220050643

[B11] Balint-Kurti PJ Clendennen SK Doleželová M Valárik M Doležel J Beetham PR May GD Identification and chromosomal localization of the *monkey *retrotransposon in *Musa *sp Mol Gen Genet 2000 263 908 915 10.1007/s004380000265 10954075

[B12] Cheung F Town CD A BAC end view of the *Musa acuminata *genome BMC Plant Biol 2007 7 29 10.1186/1471-2229-7-29 17562019 PMC1904220

[B13] Global *Musa *Genomics Consortium (GMGC) http://www.musagenomics.org/

[B14] Margulies M Egholm M Altman WE Attiya S Bader JS Bemben LA Berka J Braverman MS Chen YJ Chen ZT Dewell SB Du L Fierro JM Gomes XV Godwin BC He W Helgesen S Ho CH Irzyk GP Jando SC Alenquer MLI Jarvie TP Jirage KB Kim JB Knight JR Lanza JR Leamon JH Lefkowitz SM Lei M Li J Lohman KL Lu H Makhijani VB McDade KE McKenna MP Myers EW Nickerson E Nobile JR Plant R Puc BP Ronan MT Roth GT Sarkis GJ Simons JF Simpson JW Srinivasan M Tartaro KR Tomasz A Vogt KA Volkmer GA Wang SH Wang Y Weiner MP Yu PG Begley RF Rothberg JM Genome sequencing in microfabricated high-density picolitre reactors Nature 2005 473 376 380 10.1038/nature03959 PMC1464427 16056220

[B15] Macas J Neumann P Navrátilová A Repetitive DNA in the pea (*Pisum sativum *L.) genome: comprehensive characterization using 454 sequencing and comparison to soybean and *Medicago truncatula* BMC Genomics 2007 8 427 10.1186/1471-2164-8-427 18031571 PMC2206039

[B16] Swaminathan K Varala K Hudson ME Global repeat discovery and estimation of genomic copy number in a large, complex genome using a high-throughput 454 sequence survey BMC Genomics 2007 8 132 10.1186/1471-2164-8-132 17524145 PMC1894642

[B17] Aert R Sagi L Volckaer G Gene content and density in banana (*Musa acuminata*) as revealed by genomic sequencing of BAC clones Theor Appl Genet 2004 109 129 139 10.1007/s00122-004-1603-2 14985976

[B18] Azhar M Heslop-Harrison JS Genomes, diversity and resistance gene analogues in *Musa *species Cytogenet Genome Res 2008 121 59 66 10.1159/000124383 18544928

[B19] Vilarinhos AD Piffanelli P Lagoda P Thibivilliers S Sabau X Carreel F DHont A Construction and characterization of a bacterial artificial chromosome library of banana (*Musa acuminata *Colla) Theor Appl Genet 2003 106 1102 1106 12671759 10.1007/s00122-002-1155-2

[B20] Pillay M Ssebuliba R Hartman J Vuylsteke D Talengera D Tushemereirwe W Conventional breeding strategies to enhance the sustainability of *Musa *biodiversity conservation for endemic cultivars African Crop Science Journal 2004 12 59 65

[B21] Moens T Sandoval Fernandez JA Escalant JV De Waele D Evaluation of the progeny from a cross between 'Pisang Berlin' and *M. acuminata *spp. *burmannicoides *'Calcutta 4' for evidence of segregation with respect to resistance to black leaf streak disease and nematodes Infomusa 2002 11 20 22

[B22] Bartoš J Paux E Kofler R Havránková M Kopecký D Suchánková P Šafář J Šimková H Town CD Lelley T Feuillet C Doležel J A first survey of the rye (*Secale cereale*) genome composition through BAC end sequencing of the short arm of chromosome 1R BMC Plant Biol 2008 8 95 10.1186/1471-2229-8-95 18803819 PMC2565679

[B23] International Rice Genome Sequencing Project The map-based sequence of the rice genome Nature 2005 436 793 800 10.1038/nature03895 16100779

[B24] Velasco R Zharkikh A Troggio M Cartwright DA Cestaro A Pruss D Pindo M FitzGerald LM Vezzulli S Reid J Malacarne G Iliev D Coppola G Wardell B Micheletti D Macalma T Facci M Mitchell JT Perazzolli M Eldredge G Gatto P Oyzerski R Moretto M Gutin N Stefanini M Chen Y Segala C Davenport C Dematte L Mraz A Battilana J Stormo K Costa F Tao QZ Si-Ammour A Harkins T Lackey A Perbost C Taillon B Stella A Fawcett JA Sterck L Vandepoele K Grando SM Toppo S Moser C Lanchbury J Bogden R Skolnick M Sgaramella V Bhatnagar SK Fontana P Gutin A Van de Peer Y Salamini F Viola R High quality draft consensus sequence of the genome of a heterozygous grapevine variety PLoS ONE 2007 2 12 e1326 10.1371/journal.pone.0001326 18094749 PMC2147077

[B25] Marin I Llorens C *Ty3/Gypsy *retrotransposons: Description of a new *Arabidopsis thaliana *elements and evolutionary perspectives derived from comparative genomics data Mol Biol Evol 2000 17 1040 1049 10889217 10.1093/oxfordjournals.molbev.a026385

[B26] Havecker ER Gao X Voytas DF The Sireviruses, a plant-specific lineage of the Ty1/*copia *retrotransposons, interact with a family of proteins related to dynein light chain 8 Plant Physiol 2005 139 857 868 10.1104/pp.105.065680 16183843 PMC1256001

[B27] Wicker T Keller B Genome-wide comparative analysis of *copia *retrotransposon in triticeae, rice, and *Arabidopsis *reveals conserved ancient evolutionary lineages and distinct dynamics of individual *copia *families Genome Res 2007 17 1072 1081 10.1101/gr.6214107 17556529 PMC1899118

[B28] Grandbastien M-A Spielmann A Caboche M Tnt1, a mobile retroviral-like transposable element of tobacco isolated by plant cell genetics Nature 1989 337 376 380 10.1038/337376a0 2536143

[B29] White SE Habera LF Wessler SR Retrotransposons in the flanking region of normal plant genes: A role for *copia*-like elements in the evolution of gene structure and expression Proc Natl Acad Sci USA 1994 91 11792 11796 10.1073/pnas.91.25.11792 7991537 PMC45321

[B30] Gorinsek B Gubensek F Kordis D Evolutionary genomics of chromoviruses in eukaryotes Mol Biol Evol 2004 21 781 798 10.1093/molbev/msh057 14739248

[B31] Kordis D A genomic perspective on the chromodomain-containing retrotransposons: Chromoviruses Gene 2005 347 161 173 10.1016/j.gene.2004.12.017 15777633

[B32] Llorens C Fares MA Moya A Relationships of gag-pol diversity between *Ty3/Gypsy *and *Retroviridae *LTR retroelements and the three kings hypothesis BMC Evol Biol 2008 8 276 10.1186/1471-2148-8-276 18842133 PMC2577118

[B33] Schmidt T LINEs, SINEs and repetitive DNA: non-LTR retrotransposons in plant genomes Plant Mol Biol 1999 40 903 910 10.1023/A:1006212929794 10527415

[B34] Rubin E Lithwick G Levy AA Structure and evolution of the hAT transposon superfamily Genetics 2001 158 949 957 11454746 10.1093/genetics/158.3.949 PMC1461711

[B35] Messing J Bharti AK Karlowski WM Gundlach H Kim HR Yu Y Wei F Fuks G Soderlund CA Mayer KF Wing RA Sequence composition and genome organization of maize Proc Natl Acad Sci USA 2004 101 14349 14354 10.1073/pnas.0406163101 15388850 PMC521949

[B36] Raap AK Florijn RJ Blonden LJ Wiegant J Vaandrager JW Vrolijk H den Dunnen J Tanke HJ Ommen GJ Fiber FISH as a DNA mapping tool Methods 1996 9 67 73 10.1006/meth.1996.0009 9245344

[B37] Pedersen C Linde-Laursen I The relationship between physical and genetic distances at the *Hor1 *and *Hor2 *loci of barley estimated by two-colour fluorescent in situ hybridization Theor Appl Genet 1995 91 941 946 10.1007/BF00223904 24169981

[B38] Teo CH Schwarzacher T Tandem repeats and *Musa *chromosome organisation Unpublished GenBank code: AM909712 - AM909714

[B39] Galasso I Schmidt T Pignone D Heslop-Harrison JS The molecular cytogenetics of *Vigna unguiculata *(L.) Walp: the physical organization and characterization of 18*s*-5.8*s*-25*s *rRNA genes, 5*s *rRNA genes, telomere-like sequences, and a family of centromeric repetitive DNA sequences Theor Appl Genet 1995 91 928 935 10.1007/BF00223902 24169979

[B40] Han YH Zhang ZH Liu JH Lu JY Huang SW Jin WW Distribution of the tandem repeat sequences and karyotyping in cucumber (*Cucumis sativus *L.) by fluorescence in situ hybridization Cytogenet Genome Res 2008 122 80 88 10.1159/000151320 18931490

[B41] Jiang J Birchler JA Parrott WA Dawe RK A molecular view of plant centromeres Trends Plant Sci 2003 8 570 574 10.1016/j.tplants.2003.10.011 14659705

[B42] Nagaki K Tsujimoto H Sasakuma T A novel repetitive sequence of sugar cane, SCEN family, locating on centromeric regions Chromosome Res 1998 6 295 302 10.1023/A:1009270824142 9688519

[B43] Paux E Roger D Badaeva E Gay G Bernard M Sourdille P Feuillet C Characterizing the composition and evolution of homoeologous genomes in hexaploid wheat through BAC-end sequencing on chromosome 3B Plant J 2006 48 463 747 10.1111/j.1365-313X.2006.02891.x 17010109

[B44] Laboratory of Molecular Cytogenetics and Cytomnetry (IEB, Czech Republic) http://olomouc.ueb.cas.cz/banana-sequencing-data

[B45] Macas J Pech J Novák P PROFREP: a web server for repeat detection in genomic sequences based on 454 sequencing data http://w3lamc.umbr.cas.cz/profrep/public/

[B46] Zhang HB Zhao X Ding X Paterson AH Wing RA Preparation of megabase-size DNA from plant nuclei Plant J 1995 7 175 184 10.1046/j.1365-313X.1995.07010175.x

[B47] Pertea G Huang X Liang F Antonescu V Sultana R Karamycheva S Lee Y White J Cheung F Parvizi B Tsai J Quackenbush J TIGR Gene Indices clustering tools (TGICL): a software system for fast clustering of large EST datasets Bioinformatics 2003 19 651 652 10.1093/bioinformatics/btg034 12651724

[B48] Altschul SF Madden TL Schäffer AA Zhang J Zhang Z Miller W Lipman DJ Gapped BLAST and PSI-BLAST: a new generation of protein database search programs Nucleic Acids Res 1997 25 3389 3402 10.1093/nar/25.17.3389 9254694 PMC146917

[B49] Marchler-Bauer A Anderson JB DeWeese-Scott C Fedorova ND Geer LY He S Hurwitz DI Jackson JD Jacobs AR Lanczycki CJ Liebert CA Liu C Madej T Marchler GH Mazumder R Nikolskaya AN Panchenko AR Rao BS Shoemaker BA Simonyan V Song JS Thiessen PA Vasudevan S Wang Y Yamashita RA Yin JJ Bryant SH CDD: a curated Entrez database of conserved domain alignments Nucleic Acids Res 2003 31 383 387 10.1093/nar/gkg087 12520028 PMC165534

[B50] Sonnhammer ELL Durbin R A dot-matrix program with dynamic threshold control suited for genomic DNA and protein sequence analysis Gene 1995 167 GC1 GC10 10.1016/0378-1119(95)00714-8 8566757

[B51] Thompson JD Gibson TJ Plewniak F Jeanmougin F Higgins DG The CLUSTAL_X windows interface: flexible strategies for multiple sequence alignment aided by quality analysis tools Nucleic Acids Res 1997 25 4876 4882 10.1093/nar/25.24.4876 9396791 PMC147148

[B52] Benson G Tandem repeats finder: a program to analyze DNA sequences Nucleic Acids Res 1999 27 573 580 10.1093/nar/27.2.573 9862982 PMC148217

[B53] Sobreira TJP Durham AM Gruber A TRAP:automated classification, quantification and annotation of tandemly repeated sequences Bioinformatics 2006 22 361 362 10.1093/bioinformatics/bti809 16332714

[B54] Paux E Faure S Choulet F Roger D Gauthier V Martinant JP Sourdille P Balfourier F Le Paslier M-C Chauveau A Cakir M Gandon B Feuillet C Insertion site-based polymorphism markers open new perspectives for genome saturation and marker-assisted selection in wheat Plant Biotechnol J 2010 8 196 210 10.1111/j.1467-7652.2009.00477.x 20078842

[B55] Staden R The Staden sequence analysis package Mol Biotechnol 1996 5 233 241 10.1007/BF02900361 8837029

[B56] Doleželová M Valárik M Swennen R Horry JP Doležel J Physical mapping of the 18S-25S and 5S ribosomal RNA genes in diploid bananas Biol Plantarum 1998 41 497 505 10.1023/A:1001880030275

