# Supplementary material for: Repetitive part of the banana (Musa acuminata) genome investigated by low-depth 454 sequencing
Source: BMC Plant Biol. 2010 Sep 16;10:204. doi: 10.1186/1471-2229-10-204 (PMC2956553; doi:10.1186/1471-2229-10-204)
Supplement: Additional file 1 — Genome proportion of newly characterized banana repetitive elements. Genome proportion of repetitive elements was estimated from the sum of genome representation values (GR) of corresponding clusters of reads. [file 1471-2229-10-204-S1.PDF]

Additional file 1: Genome representation of DNA repeats in banana nuclear genome.

	Genome Representation		
	Number of clusters (contigs)	Total GR	Genome proportion [%]
Retroelements			
Ty1/copia			16.94
SIRE/Maximus evolutionary lineage	7 (727)	11770995	12.60
Angela evolutionary lineage	4 (178)	2789274	2.98
Tnt1 evolutionary lineage	3 (64)	1081887	1.16
Hopscotch evolutionary lineage	2 (3)	12263	0.01
Unclassified Ty1/copia	13 (24)	204117	0.19
Ty3/gypsy			7.52
Chromoviruses evolutionary lineage			
Reina clade	2 (127)	3699993	3.95
Tekay clade	3 (145)	1686968	1.82
CRM clade	1 (18)	389613	0.42
Galadriel clade	1 (22)	366130	0.39
Tat evolutionary lineage	2 (2)	5653	0.01
Unclassified Ty3/gypsy	4 (55)	894034	0.93
non-LTR			1.00
LINE	1 (35)	933556	1.00
Unclassified RE	8 (186)	1960707	2.09
DNA transposons	2 (16)	84087	0.09
Tandem repeats		1655390	1.79
45SrDNA	1 (13)	1046074	1.12
5SrDNA	1 (6)	355695	0.38
Other satellites	3 (19)	253621	0.29
