# Supplementary material for: Repetitive part of the banana (Musa acuminata) genome investigated by low-depth 454 sequencing
Source: BMC Plant Biol. 2010 Sep 16;10:204. doi: 10.1186/1471-2229-10-204 (PMC2956553; doi:10.1186/1471-2229-10-204)
Supplement: Additional file 2 — Repetitive profiles of sequenced BAC clones. DNA sequence profiles of selected clones from three BAC libraries of M. acuminata cv. 'Calcutta 4' (MA4 and C4BAM), M. acuminata cv. 'Cavendish' (MAC) and MBP BAC library from M. balbisiana cv. 'Pisang Klutug Wulung' http://olomouc.ueb.cas.cz/dna-libraries/bananas. [file 1471-2229-10-204-S2.PDF]

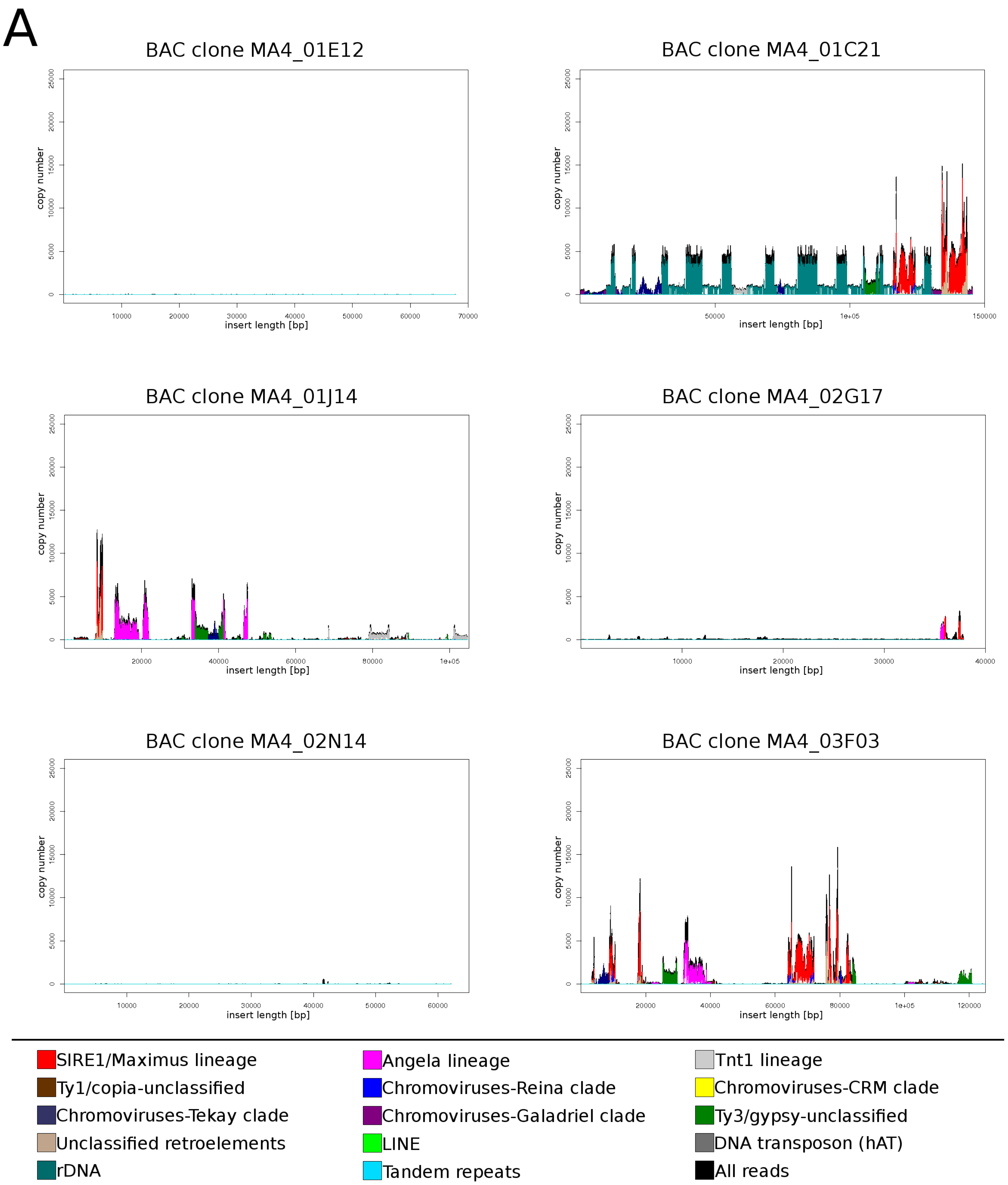
A
BAC clone MA4_01E12
BAC clone MA4_01C21
BAC clone MA4_01J14
BAC clone MA4_02G17
BAC clone MA4_02N14
BAC clone MA4_03F03
copy number
insert length [bp]
SIRE1/Maximus lineage
Ty1/copia-unclassified
Chromoviruses-Tekay clade
Unclassified retroelements
rDNA
Angela lineage
Chromoviruses-Reina clade
Chromoviruses-Galadriel clade
LINE
Tandem repeats
Tnt1 lineage
Chromoviruses-CRM clade
Ty3/gypsy-unclassified
DNA transposon (hAT)
All reads

B

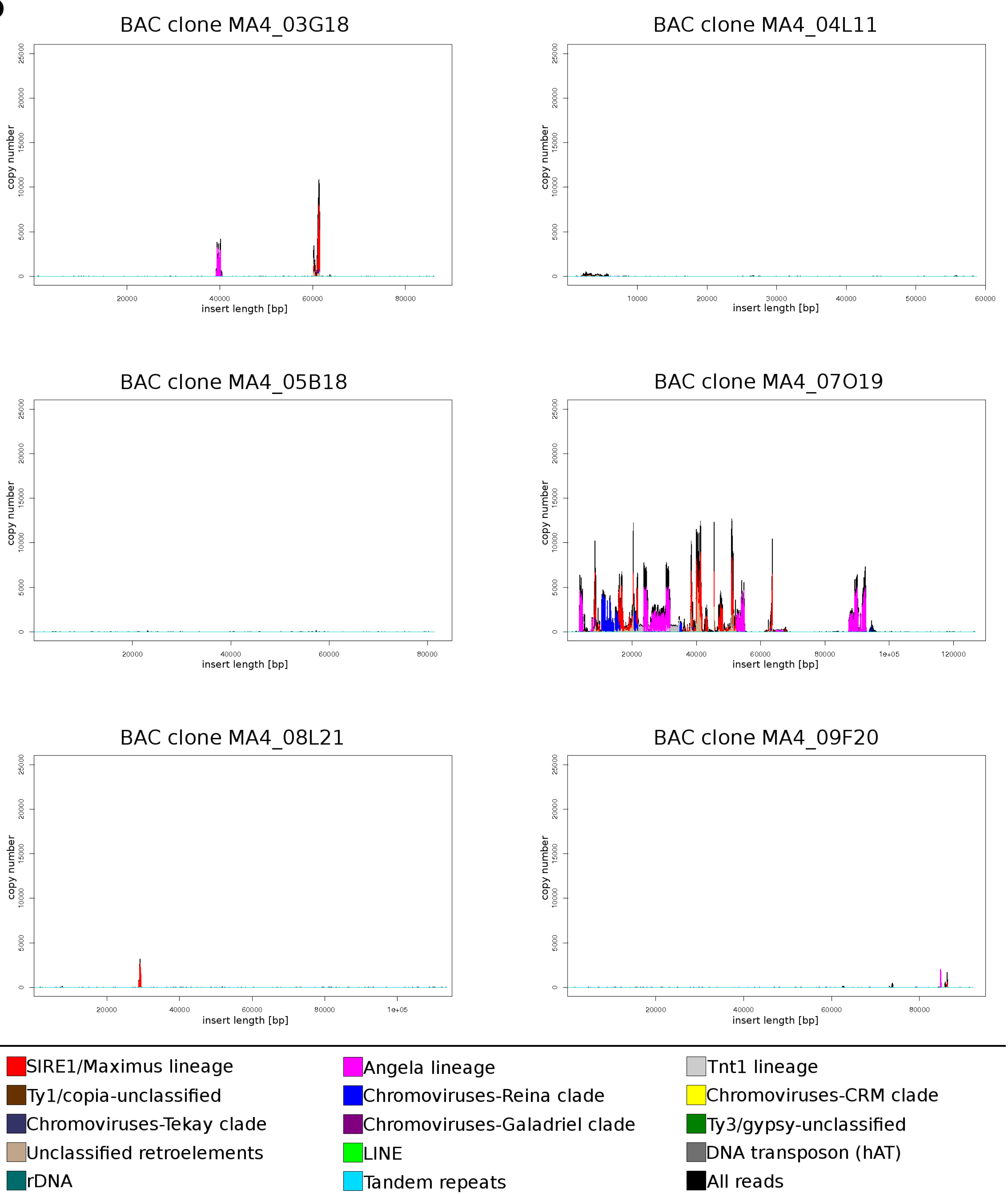
BAC clone MA4_03G18
BAC clone MA4_04L11
BAC clone MA4_05B18
BAC clone MA4_07O19
BAC clone MA4_08L21
BAC clone MA4_09F20
copy number
insert length [bp]
SIRE1/Maximus lineage
Angela lineage
Tnt1 lineage
Ty1/copia-unclassified
Chromoviruses-Reina clade
Chromoviruses-CRM clade
Chromoviruses-Tekay clade
Chromoviruses-Galadriel clade
Ty3/gypsy-unclassified
Unclassified retroelements
LINE
DNA transposon (hAT)
rDNA
Tandem repeats
All reads

C

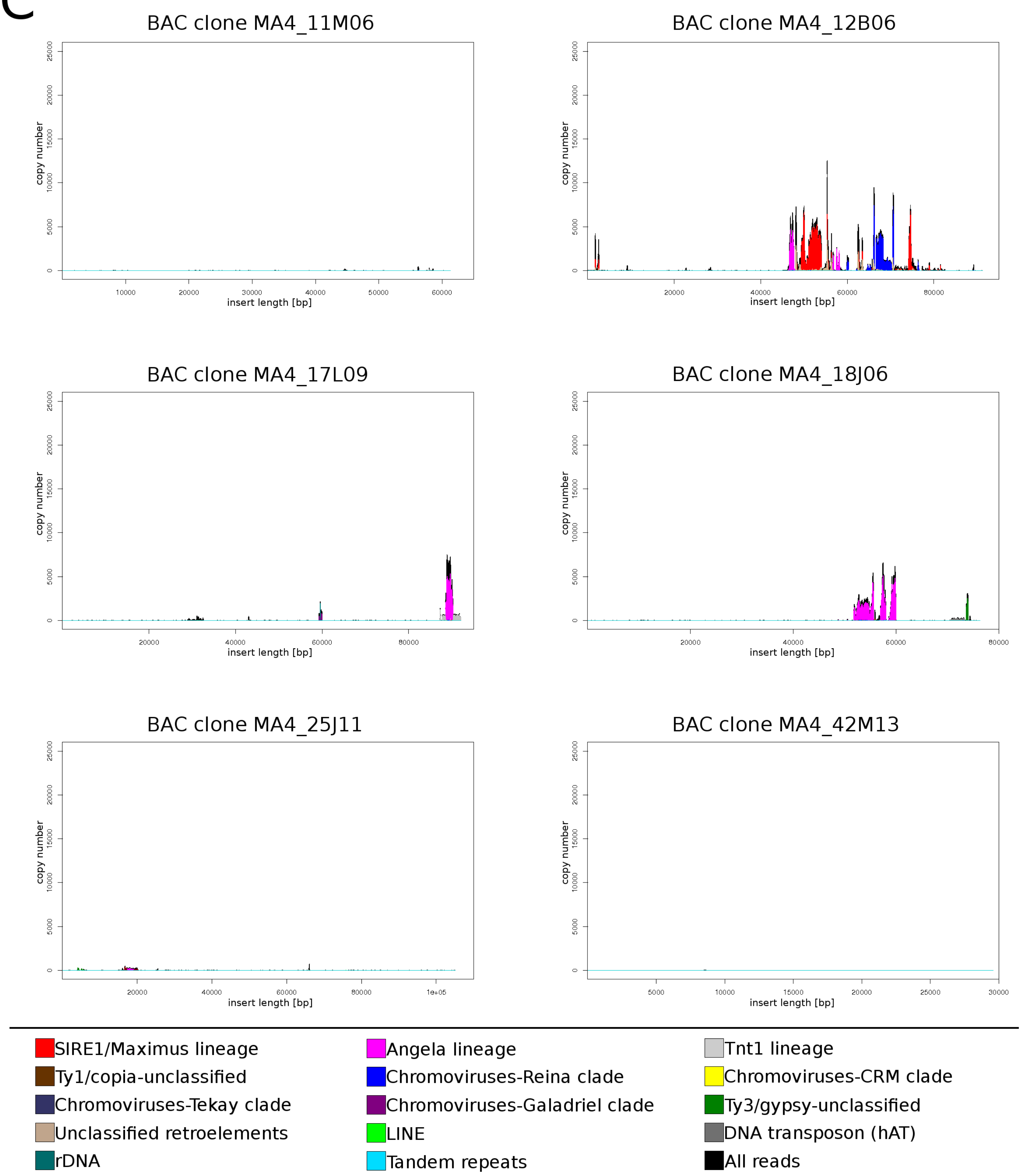
BAC clone MA4_11M06
BAC clone MA4_12B06
BAC clone MA4_17L09
BAC clone MA4_18J06
BAC clone MA4_25J11
BAC clone MA4_42M13
copy number
insert length [bp]
0
5000
10000
15000
20000
25000
30000
40000
50000
60000
80000
1e+05
SIRE1/Maximus lineage
Ty1/copia-unclassified
Chromoviruses-Tekay clade
Unclassified retroelements
rDNA
Angela lineage
Chromoviruses-Reina clade
Chromoviruses-Galadriel clade
LINE
Tandem repeats
Tnt1 lineage
Chromoviruses-CRM clade
Ty3/gypsy-unclassified
DNA transposon (hAT)
All reads

D

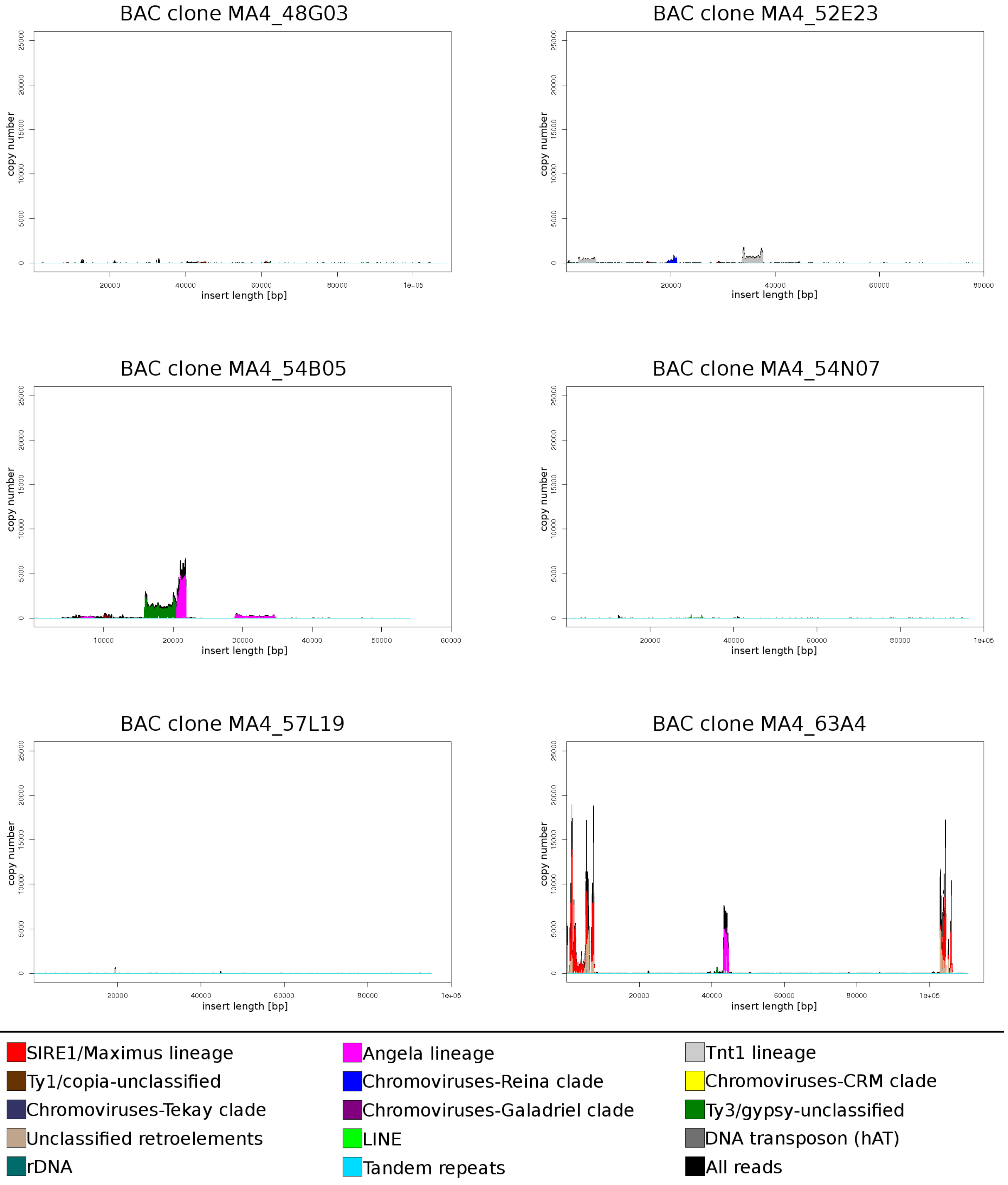
BAC clone MA4_48G03
BAC clone MA4_52E23
BAC clone MA4_54B05
BAC clone MA4_54N07
BAC clone MA4_57L19
BAC clone MA4_63A4
copy number
insert length [bp]
SIRE1/Maximus lineage
Angela lineage
Tnt1 lineage
Ty1/copia-unclassified
Chromoviruses-Reina clade
Chromoviruses-CRM clade
Chromoviruses-Tekay clade
Chromoviruses-Galadriel clade
Ty3/gypsy-unclassified
Unclassified retroelements
LINE
DNA transposon (hAT)
rDNA
Tandem repeats
All reads

E

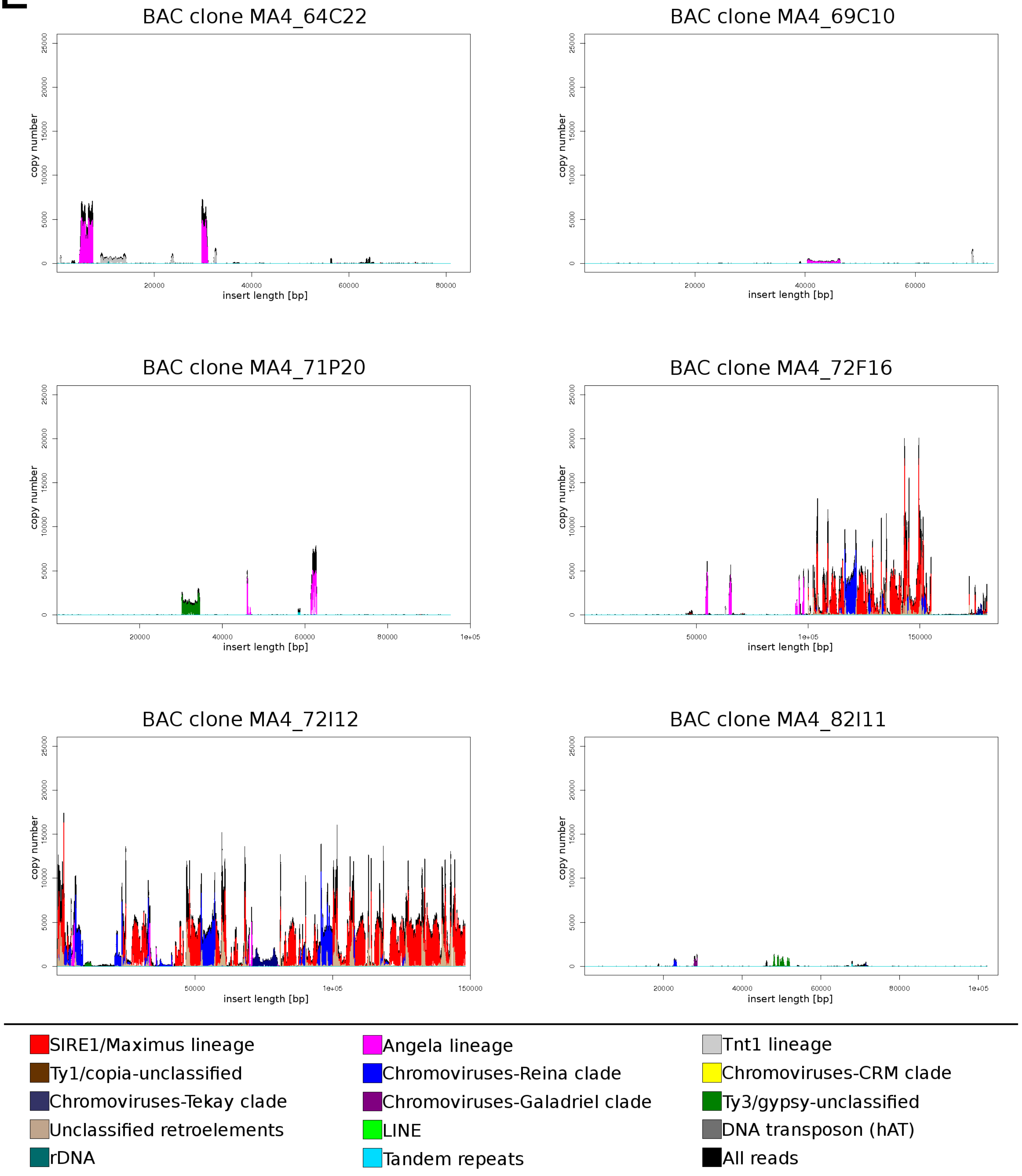

BAC clone MA4_64C22
BAC clone MA4_69C10
BAC clone MA4_71P20
BAC clone MA4_72F16
BAC clone MA4_72I12
BAC clone MA4_82I11
copy number
insert length [bp]
SIRE1/Maximus lineage
Ty1/copia-unclassified
Chromoviruses-Tekay clade
Unclassified retroelements
rDNA
Angela lineage
Chromoviruses-Reina clade
Chromoviruses-Galadriel clade
LINE
Tandem repeats
Tnt1 lineage
Chromoviruses-CRM clade
Ty3/gypsy-unclassified
DNA transposon (hAT)
All reads

F

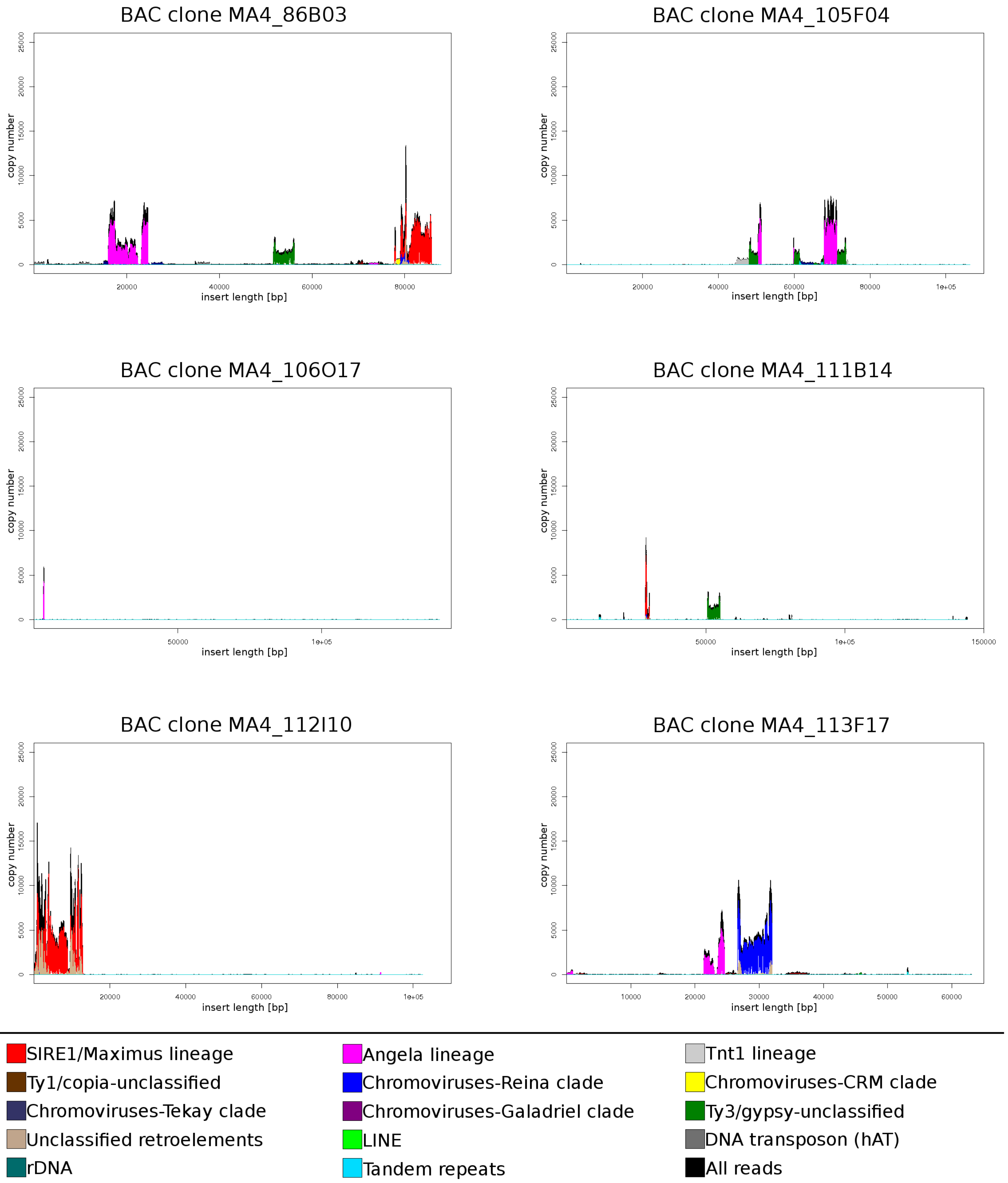
BAC clone MA4_86B03
BAC clone MA4_105F04
BAC clone MA4_106O17
BAC clone MA4_111B14
BAC clone MA4_112I10
BAC clone MA4_113F17
copy number
insert length [bp]
SIRE1/Maximus lineage
Ty1/copia-unclassified
Chromoviruses-Tekay clade
Unclassified retroelements
rDNA
Angela lineage
Chromoviruses-Reina clade
Chromoviruses-Galadriel clade
LINE
Tandem repeats
Tnt1 lineage
Chromoviruses-CRM clade
Ty3/gypsy-unclassified
DNA transposon (hAT)
All reads

G

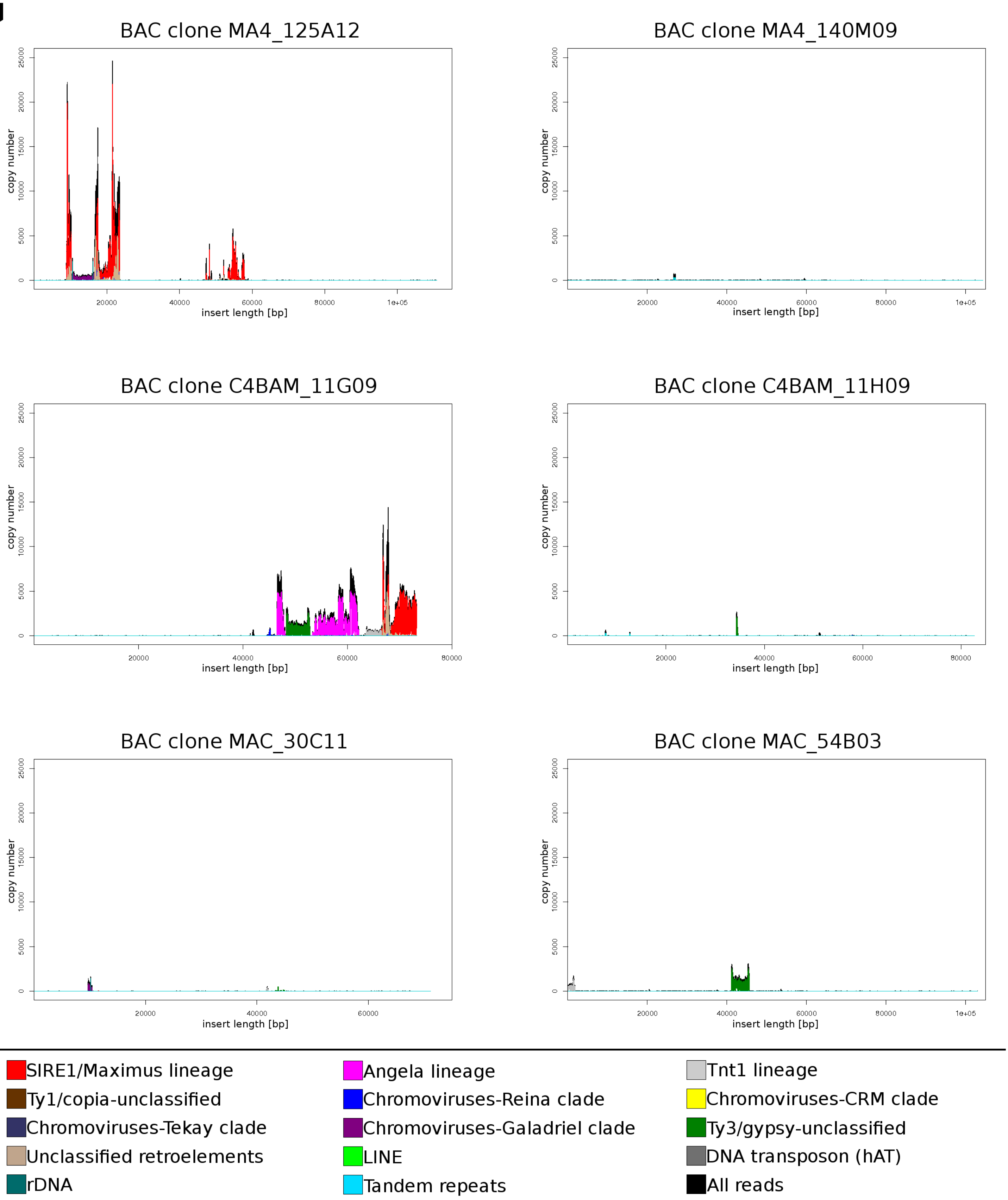
BAC clone MA4_125A12
BAC clone MA4_140M09
BAC clone C4BAM_11G09
BAC clone C4BAM_11H09
BAC clone MAC_30C11
BAC clone MAC_54B03
copy number
insert length [bp]
0
5000
10000
15000
20000
25000
20000
40000
60000
80000
1e+05
SIRE1/Maximus lineage
Ty1/copia-unclassified
Chromoviruses-Tekay clade
Unclassified retroelements
rDNA
Angela lineage
Chromoviruses-Reina clade
Chromoviruses-Galadriel clade
LINE
Tandem repeats
Tnt1 lineage
Chromoviruses-CRM clade
Ty3/gypsy-unclassified
DNA transposon (hAT)
All reads

H

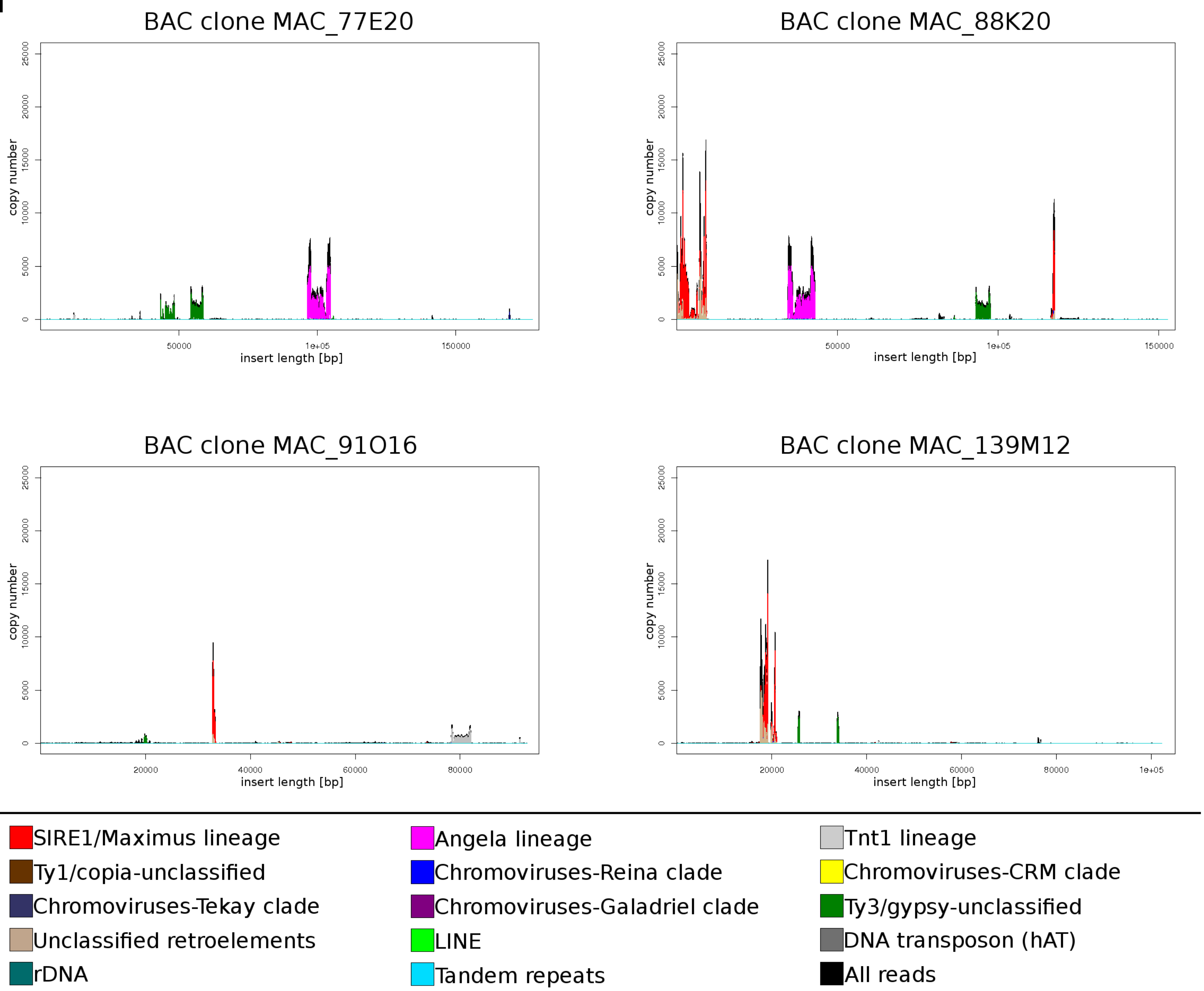
BAC clone MAC_77E20
copy number
0
5000
10000
15000
20000
25000
50000
1e+05
150000
insert length [bp]
BAC clone MAC_88K20
copy number
0
5000
10000
15000
20000
25000
50000
1e+05
150000
insert length [bp]
BAC clone MAC_91O16
copy number
0
5000
10000
15000
20000
25000
20000
40000
60000
80000
insert length [bp]
BAC clone MAC_139M12
copy number
0
5000
10000
15000
20000
25000
20000
40000
60000
80000
1e+05
insert length [bp]
SIRE1/Maximus lineage
Ty1/copia-unclassified
Chromoviruses-Tekay clade
Unclassified retroelements
rDNA
Angela lineage
Chromoviruses-Reina clade
Chromoviruses-Galadriel clade
LINE
Tandem repeats
Tnt1 lineage
Chromoviruses-CRM clade
Ty3/gypsy-unclassified
DNA transposon (hAT)
All reads

I

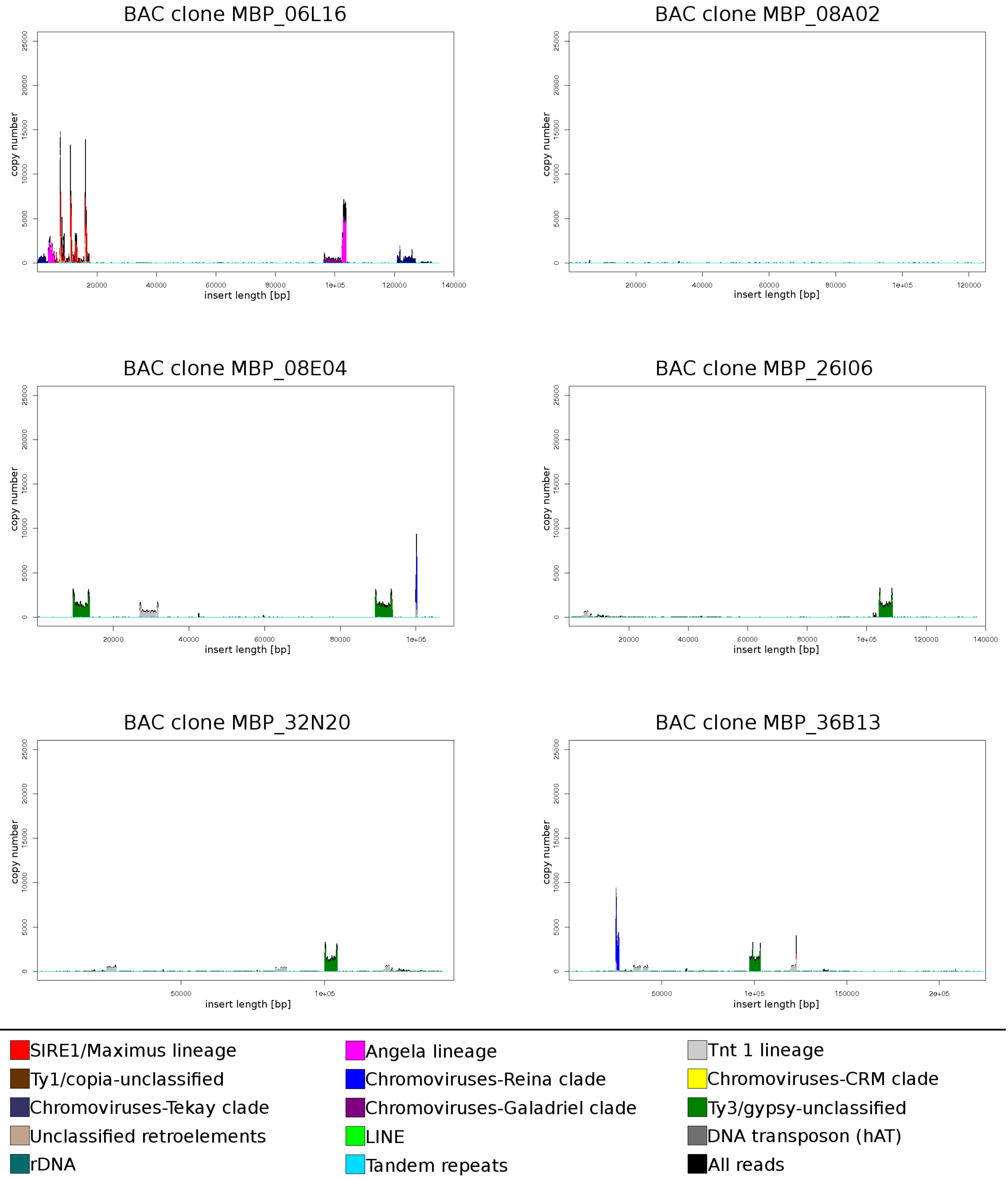
BAC clone MBP_06L16
BAC clone MBP_08A02
BAC clone MBP_08E04
BAC clone MBP_26I06
BAC clone MBP_32N20
BAC clone MBP_36B13
copy number
insert length [bp]
0
5000
10000
15000
20000
25000
20000
40000
60000
80000
1e+05
120000
140000
50000
150000
2e+05
SIRE1/Maximus lineage
Ty1/copia-unclassified
Chromoviruses-Tekay clade
Unclassified retroelements
rDNA
Angela lineage
Chromoviruses-Reina clade
Chromoviruses-Galadriel clade
LINE
Tandem repeats
Tnt 1 lineage
Chromoviruses-CRM clade
Ty3/gypsy-unclassified
DNA transposon (hAT)
All reads

J

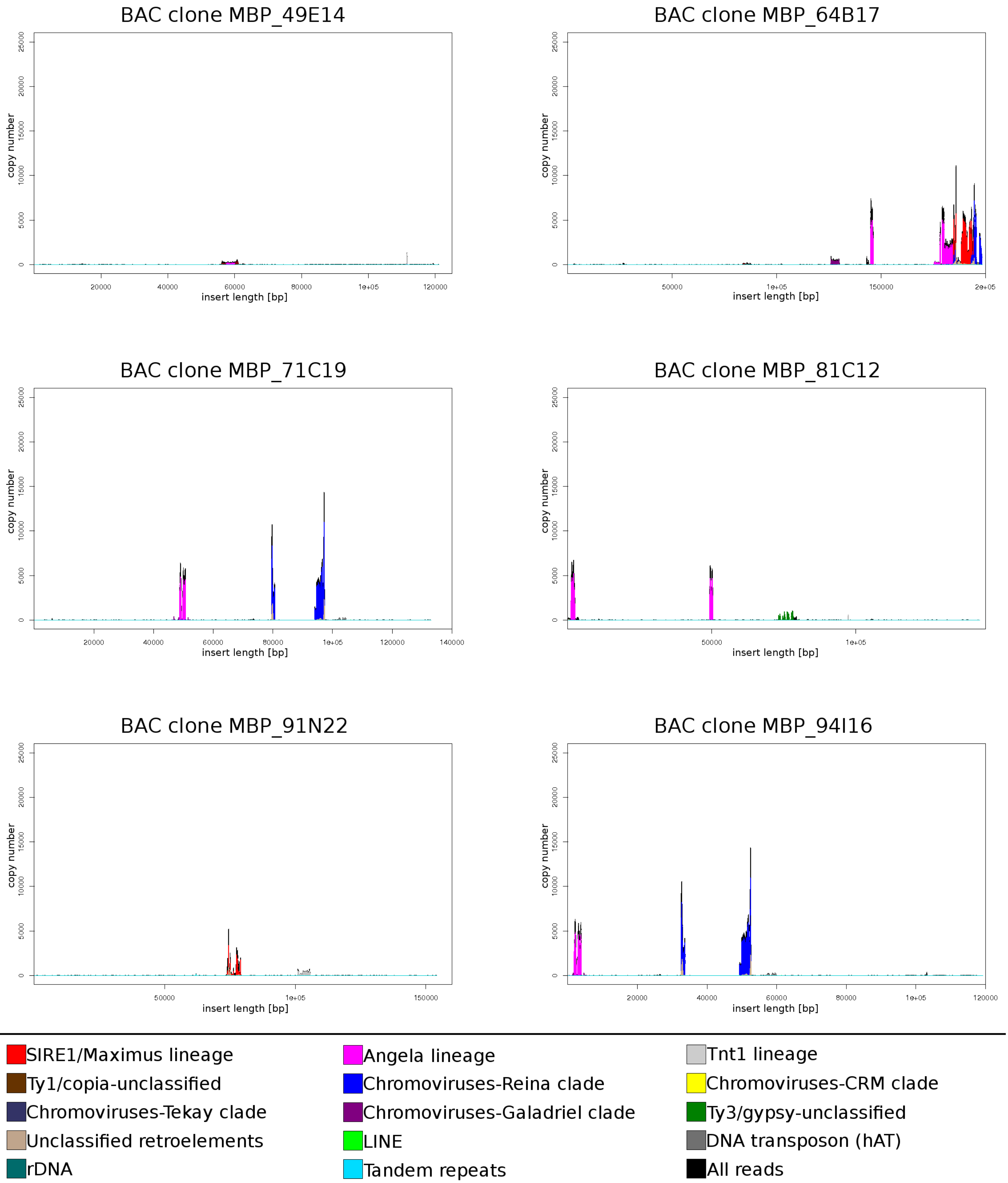
BAC clone MBP_49E14
BAC clone MBP_64B17
BAC clone MBP_71C19
BAC clone MBP_81C12
BAC clone MBP_91N22
BAC clone MBP_94I16
copy number
insert length [bp]
SIRE1/Maximus lineage
Ty1/copia-unclassified
Chromoviruses-Tekay clade
Unclassified retroelements
rDNA
Angela lineage
Chromoviruses-Reina clade
Chromoviruses-Galadriel clade
LINE
Tandem repeats
Tnt1 lineage
Chromoviruses-CRM clade
Ty3/gypsy-unclassified
DNA transposon (hAT)
All reads
