# Supplementary material for: Repetitive part of the banana (Musa acuminata) genome investigated by low-depth 454 sequencing
Source: BMC Plant Biol. 2010 Sep 16;10:204. doi: 10.1186/1471-2229-10-204 (PMC2956553; doi:10.1186/1471-2229-10-204)
Supplement: Additional file 3 — Primers used for PCR amplification of satellite DNA and different types of retrotransposons. [file 1471-2229-10-204-S3.PDF]

Additional file 3 – Primers used for PCR amplification of satellite DNA and different types of retrotransposons

Contig name	Repetitive DNA sequences	Primer sequence
	**Ty1/copia**	
CL1SCL2Contig945	SIRE/Maximus evolutionary lineage	5'-CATGCAGTGTTTACGGCTTC<br>5'-CGCCTTTGTCATTGTTGATG
CL1SCL2Contig1080		5'-TCCTCGGAGTGGAGAAGTTG<br>5'-TGCTTTTGTGATTGTGGATGA
CL2Contig49	Angela evolutionary lineage	5'-GGAGGTGTCCATTGGGATAAA<br>5'-AACTCATGCCATTGGTGGTT
CL21Contig3		5'-TTTCTGCAACACCATTCTGG<br>5'-CATTGGATGCCTTCAAAGTCT
CL10Contig16	Tnt1 evolutionary lineage	5'-ACATGCTCTGCAACATCACC<br>5'-TGGGATCCAACATGAGATGA
CL12Contig16		5'-GCTCCCAATACTGGTCTCCA<br>5'-TGGAAGCGAGAACAGAATGA
	**Ty3/gypsy**	
	Chromoviruses evolutionary lineage	
CL1SCL5Contig891	Reina clade	5'-CTAGCGAAACACCGGAGGTA<br>5'-GCTGCCATCTTCCAAAGGTA
CL4Contig82	Tekay clade	5'-CCATTCACCTTTCCAATCCA<br>5'-TCTGCACATTTCCTACCGATT
CL11Contig14	CRM clade	5'-AATTTCCCAAGCTTCGGTTT<br>5'-CTCCATGGGAGGATGTGAGT
	**Non-LTR**	
CL1SCL8Contig452	LINE	5'-TGAAAGCAGCTTGATTTGGA<br>5'-CAAGGCTTGCCAACATTTTT
CL25Contig7	**DNA transposons (hAT)**	5'-TGCTTCCATTGTCGGTTATG<br>5'-GAGATGCGAGTTCCATTGCT
CL18Contig7	**Tandem repeat**	5'-TGCATTATGCGTTGTCATTATTT<br>5'-TCTAACCCGCTCTCACGAAT
CL33Contig3	**Tandem repeat**	5'-CGTATATGCTGGGCTTCGTT<br>5'-CACACTCCCGTGCCTTTAAT
